# Prostate-specific antigen-based screening: controversy and guidelines

**DOI:** 10.1186/s12916-015-0296-5

**Published:** 2015-03-24

**Authors:** Eric H Kim, Gerald L Andriole

**Affiliations:** Washington University School of Medicine, 4960 Children’s Place, Campus Box 8242, St. Louis, MO 63110 USA

**Keywords:** Prostate-specific antigen, PSA, Prostate cancer, Cancer screening, Cancer screening tests, Active surveillance, Watchful waiting, Prostatectomy, Guidelines

## Abstract

Although prostate-specific antigen (PSA) screening has improved the detection of prostate cancer, allowing for stage migration to less advanced disease, the precise mortality benefit of early detection is unclear. This is in part due to a discrepancy between the two large randomized controlled trials comparing PSA screening to usual care. The European Randomized Study of Screening for Prostate Cancer (ERSPC) found a survival benefit to screening, while the United States Prostate, Lung, Colorectal, and Ovarian (PLCO) Cancer Screening Trial did not. Furthermore, the benefit of immediate surgical intervention for screen-detected prostate cancer is unclear, as the results superficially differ between the two large randomized controlled trials comparing prostatectomy to observation. The Prostate Cancer Intervention Versus Observation Trial (PIVOT) found no survival benefit for prostatectomy in PSA screened U.S. men, while the Scandinavian Prostate Cancer Group Study Number Four (SPCG-4) found a survival benefit for prostatectomy in clinically diagnosed prostate cancer. As a result of the controversy surrounding PSA screening and subsequent prostate cancer treatment, guidelines vary widely by organization.

## Background

In 1991 Catalona and colleagues first reported the use of prostate-specific antigen (PSA) for prostate cancer (PCa) screening [1]. The adoption of PSA screening in the United States dramatically increased the detection of PCa, particularly organ-confined disease [2-4]. Between 1986 and 1993, the incidence of PCa increased from 86 to 179 cases per 100,000 white men and 124 to 250 cases per 100,000 black men. However, the rate of distant disease at the time of diagnosis fell from 14.9 to 6.6 cases per 100,000 men during the same time period [5].

Some feared that PSA screening would contribute to overdiagnosis and subsequent overtreatment of PCa, with potential net unfavorable effects on patient mortality and quality of life [6]. This was in part due to observations that men with localized, low-grade PCa had low disease-specific ten-year mortality, and those with life expectancy less than ten years experienced no change in survival with conservative management [7-9]. In order to accurately assess the benefit of PSA screening, the United States Prostate, Lung, Colorectal, and Ovarian (PLCO) Cancer Screening Trial and the European Randomized Study of Screening for Prostate Cancer (ERSPC) were conceived [10,11].

The discordant results of these studies have led to further controversy regarding PSA screening, evidenced by the differences among various PCa screening guidelines [12]. We briefly present the findings and limitations of the studies that have contributed to this controversy, as well as summarize the various PSA screening recommendations.

## No benefit with PSA screening in the United States

From 1993 to 2001, the PLCO screening trial randomly assigned 76,693 men aged 55 to 74 years to annual PSA screening or usual care. Annual PSA testing was offered for six years, and screening also included digital rectal examination (DRE). Exclusion criteria included history of PCa and more than one PSA test in the three years prior to randomization [13].

After 13-year follow-up, the incidence of PCa was significantly higher in the screening arm (relative increase of 12%). However, the rate of PCa death was very low in both arms (3.7 versus 3.4 deaths per 10,000 person-years), and the difference was not statistically significant [14].

Explanations for the lack of mortality reduction seen with PSA screening in the PLCO trial include: 1) contamination of the control group, as 40 to 52% of patients in the usual care arm received PSA screening; 2) elimination of PCa cases prior to randomization, as 44% of patients had undergone one or more PSA tests prior to randomization; 3) no PSA threshold for biopsy (PSA results were reported to primary care physicians and a “community standard” for biopsy was applied at various centers), while the ERSPC authors used PSA cutoffs of 2.5 and 3.0 ng/mL, which was likely more sensitive.

## Survival benefit with PSA screening in Europe

From 1994 to 2000, the ERSPC trial randomly assigned 182,160 men aged 50 to 74 years to PSA screening at an average of once every four years or no screening. PSA testing was offered every four years at six out of seven centers and every two years in Sweden. A PSA value ≥3.0 ng/mL was an indication for biopsy at most centers. Patients with a history of PCa diagnosis were excluded [15].

At 11-year follow-up, the incidence of PCa was significantly higher in the screening arm (rate ratio 1.63), and the rate of PCa death was significantly reduced in the screening arm (rate ratio 0.79), with the number needed to screen to prevent one PCa death (NNS) equal to 1,055 men [16]. At 13-year follow-up, PSA screening demonstrated further mortality reduction, with NNS declining to 781 men [17].

The limitation of the ERSPC is the heterogeneity of data due to multiple centers using different screening intervals and PSA thresholds for biopsy. The greatest benefit to PSA screening was realized in the Swedish arm of ERSPC, the Goteborg trial, where 20,000 men were randomized to invitation to biannual PSA screening versus no invitation. At 14-year follow-up, the screening group had significant reductions in PCa death (rate ratio 0.56, NNS 293) [18]. However, other centers showed no significant benefit from PSA screening, including Finland, which had the largest enrollment of the ERSPC sites and very high overall PCa incidence and mortality [19].

## Effective screening requires an effective treatment: prostatectomy versus observation

The Prostate Cancer Intervention Versus Observation Trial (PIVOT) randomly assigned 731 men with localized PCa, diagnosed with PSA screening, to prostatectomy or observation with delayed androgen deprivation therapy (ADT). At 12-year follow-up, the prostatectomy group had no significant overall or PCa-specific survival benefit [20].

The Scandinavian Prostate Cancer Group Study Number Four (SPCG-4) randomly assigned 695 men with localized PCa, diagnosed by DRE or on transurethral resection specimen, to prostatectomy or watchful waiting with delayed ADT. At 15-year follow-up, the prostatectomy group had both a significant overall and PCa-specific survival benefit compared to observation, with the number needed to treat to prevent one PCa death (NNT) equal to 15 men. The benefit was confined to men younger than 65 years of age on subset analysis, with NNT equal to 7 men in this group [21].

The mortality difference between PIVOT and SPCG-4 was substantial; the relative reduction in overall mortality was 12% versus 25% and the absolute reduction in overall mortality was 2.6% versus 6.6%, respectively. This was likely a result of PIVOT enrollment of an older population (only 10% of patients were younger than 60 years of age) with less advanced PCa (50% nonpalpable versus 12% in SPCG-4). As a result, the overall rate of PCa death in PIVOT was much lower, 7.1% versus 19.6%.

## Summary of current PSA screening guidelines

PCa screening recommendations vary by organization and are summarized in Table 1. Baseline PSA testing is recommended by some groups, based on the Malmo Preventive Project, where a strong association was found between PSA at age 44 to 50 years and future diagnosis of advanced PCa [22].

**Table 1:** **Summary of PSA screening guidelines by organization**

**Organization**	**Year published**	**Baseline testing (age)**	Invitation to screening* (age)	High risk groups** (age)	**Screening interval**	**PSA threshold for biopsy (ng/mL)**
American Cancer Society [23]	2010	None	Beginning at 50 years while life expectancy ≥ 10 years	Beginning at 40 years while life expectancy ≥ 10 years	- Annually if PSA ≥ 2.5 ng/mL	- 2.5 ng/mL in select patients
					- Every 2 years if PSA < 2.5 ng/mL	- 4.0 ng/mL in most patients
U.S. Preventive Services Task Force [24]	2012	None	None	None	None	None
American Urological Association [25]	2013	None	55 - 69 years	40 - 69 years	Every 2 years	None specified
European Association of Urology [26]	2013	40 - 45 years	Any age while life expectancy ≥ 10 years	Any age while life expectancy ≥ 10 years	- Every 2 to 4 years if baseline PSA > 1 ng/mL	None specified
					- Every 8 years if baseline PSA ≤ 1 ng/mL	
American College of Physicians [27]	2013	None	50 - 69 years	40 - 69 years	Annually if PSA ≥ 2.5 ng/mL	None specified
National Comprehensive Cancer Network [28]	2014	45 - 49 years	50 - 70	Consider change in biopsy threshold	For 40 - 49 years:	- 3.0 ng/mL
			years		- Every 1 - 2 years if PSA > 1 ng/mL	- <3.0 ng/mL with excess risk based on multiple factors (family history, race, PSA kinetics)
			70 - 75 years if life expectancy ≥ 10 years		- Repeat at age 50 if PSA ≤ 1 ng/mL	
					For 50 - 70 years:	
					- Every 1 - 2 years	
Melbourne Consensus Statement [29]	2014	40 - 49 years	50 - 69 years	Use to better risk stratify men	None specified	None specified
			70+ years while life expectancy ≥ 10 years			

*For men who are well-informed on the risks and benefits of PSA screening.

**African American race and first-degree relatives diagnosed with PCa.

## Conclusions

The cause of discrepancy between the major PSA screening trials may be a result of differences in study design, screening protocol, and biopsy threshold: PLCO compared annual to opportunistic screening and relied on the community standard for indication to biopsy, while ERSPC compared biennial to quadrennial screening to no screening and defined a PSA cutoff for biopsy. As for the major intervention trials, PIVOT found that prostatectomy for PSA-diagnosed low-risk PCa may provide limited benefit, while SPCG-4 found that the same treatment is beneficial for younger patients with clinically diagnosed PCa. The forthcoming results (expected in 2016) of the Prostate Testing for Cancer and Treatment (ProtecT) trial should provide further insight regarding survival and quality of life for patients with localized PCa diagnosed by PSA screening randomized to active monitoring, prostatectomy, or radiotherapy [30].

Although the current guidelines regarding PSA screening differ by organization, overall they reflect the important findings of the above studies: 1) PSA screening should be offered only to men with reasonable life expectancy, 2) screening should be performed on a biennial or greater basis to minimize overdiagnosis, 3) additional data may be used to provide risk adjustments to screening interval and biopsy threshold, and, most importantly 4) the decision to undergo PSA screening should be made by both the provider and the well-informed patient after a complete discussion of the potentially limited benefit and associated harms of early PCa detection and treatment.

## Abbreviations

ACP: American College of Physicians
ACS: American Cancer Society
ADT: androgen deprivation therapy
DRE: digital rectal examination
ERSPC: European Randomized Study of Screening for Prostate Cancer
NCCN: National Comprehensive Cancer Network
NNS to prevent one prostate cancer death: number needed to screen
NNT to prevent one prostate cancer death: number needed to treat
PCa: prostate cancer
PIVOT: Prostate Cancer Intervention Versus Observation Trial
PLCO: Prostate, Lung, Colorectal, and Ovarian (Cancer Screening Trial)
ProtecT: Prostate Testing for Cancer and Treatment
PSA: prostate-specific antigen
SPCG-4: Scandinavian Prostate Cancer Group Study Number Four
USPSTF: U.S. Preventive Services Task Force
